# Variation of bispectral index in children aged 1–12 years under propofol anesthesia: an observational study

**DOI:** 10.1186/s12871-019-0815-6

**Published:** 2019-08-07

**Authors:** Fang Wang, Jianmin Zhang, Jie Yu, Muyang Tian, Xiaohuan Cui, Anshi Wu

**Affiliations:** 1 Department of Anesthesiology, Beijing Children’s Hospital, Capital Medical University, National Center for Children’s Health, No. 56 Nanlishi Road, Beijing, 100045 China; 2 Department of Anesthesiology, Beijing Chaoyang Hospital, Capital Medical University, No. 8 Gongti South Road, Chaoyang District, Beijing, 100020 China

**Keywords:** Propofol, Anesthesia: intravenous, Bispectral index, Children

## Abstract

**Background:**

The use of the bispectral index (BIS) is widespread in pediatric anesthesia, but few studies have attempted to perform a detailed evaluation of how BIS varies according to age in children under propofol anesthesia. This prospective study aimed to explore the exact relationship between BIS value and the age of 1- to 12-year-old children under propofol anesthesia.

**Methods:**

This study enrolled 165 children (1 < yr. ≤ 12), scheduled for surgery under anesthesia, and divided them into 11 age groups. Of the 165 participants, 157 completed the study protocol. All patients were anesthetized with propofol for over 30 s. An observation period of 4 min followed. BIS values were recorded at 0 (immediately after propofol injection), 30, 40, 50, 60, 90, 120, 180, and 240 s after the injection. BIS values at each time point corresponding to the 11 age groups were compared using repeated measures ANOVA.

**Results:**

BIS values significantly differed among the nine time points (*p* < 0.01) as well as among the different age groups (*p* < 0.01) after propofol administration. Post-hoc Bonferroni tests showed a difference in BIS values between groups 1–4 (1 < yr. ≤ 5) and groups 5–11(5 < yr. ≤ 12). BIS values were lower in the latter than in the former, from 50 to 240 s. The minimum BIS values in group 1 < yr. ≤ 5 and in group 5 < yr. ≤ 12 were recorded at 60 s as 49 ± 17 and 35 ± 14, respectively.

**Conclusions:**

During propofol anesthesia, the BIS values were closely related to age, which can be divided into two groups: 1 < yr. ≤ 5 and 5 < yr. ≤ 12. BIS values of the older age group were lower than those of the younger age group at the same time points.

**Trial registration:**

Registration number: chictr-roc-16008630. Registered on 12 June 2016. Retrospectively registered.

## Background

Since the implementation of the bispectral index (BIS) as the only means of monitoring the depth of sedation by the American Food and Drug Administration, its application has become increasingly widespread in pediatric anesthesia. Although the reliability of BIS is similar among adults and children over the age of 1 year [1], whether BIS can accurately monitor the depth of sedation in children remains controversial. The BIS algorithm was initially derived from electroencephalography data obtained from adults under various anesthetic conditions [2]. However, the child’s brain is different from that of an adult and is characterized by rapid development. Moreover, the Electroencephalogram (EEG) of children has different manifestations at different ages; thus, whether the BIS value can accurately reflect these changes across growth is directly related to its accuracy for monitoring sedation depth. Recent studies using various volatile and intravenous anesthetic agents have shown that BIS values are linked to the age of children [3–6]. However, most of these studies have involved children over the age of 3 years or traditional age groups; infants (1–12 months of age), toddlers (13–36 months), and children (37–144 months), and only indicated that a given BIS value was relevant for a specified age. Few studies have attempted to evaluate the details of BIS variation with age under propofol anesthesia in a pediatric population.

This prospective observational study explored the exact relationship between BIS values and children aged 1–12 years, grouped with one-year intervals, under propofol anesthesia to provide a reliable basis for clinical monitoring.

## Methods

### Ethical approval

This study (Chictr.org.cn identifier: chictr-roc-16,008,630) was approved by the Ethical Committee of Beijing Children’s Hospital, Capital Medical University, Beijing, China (Ethical Committee No 2016–67, Chairperson Prof Tianyou Wang) on 16 May 2016. Written informed consent for participation was obtained from the parents or guardians of all patients.

### Study population

In total, 165 pediatric patients requiring elective surgery, including urological, orthopedic, thoracic, and general surgery, under general anesthesia via intubation or a laryngeal mask, were initially selected. The inclusion criteria were as follows: 1<yr. ≤ 12, an American Society of Anesthesiologists (ASA) score of I or II, the intraoperative use of total intravenous anesthesia, and the provision of written informed consent by a parent or guardian. The exclusion criteria were as follows: concomitant severe systemic disease; requirement of cranial surgery; long-term use of sedative, hypnotic, or antipsychotic drugs; history of epilepsy; concomitant mental development disorders; and concomitant cardiovascular disease. We divided the included patients into 11 age groups with 15 patients in each group: group 1 (1 < yr. ≤ 2), group 2 (2 < yr. ≤ 3), group 3 (3 < yr. ≤ 4), group 4 (4 < yr. ≤ 5), group 5 (5 < yr. ≤ 6), group 6 (6 < yr. ≤ 7), group 7 (7 < yr. ≤ 8), group 8 (8 < yr. ≤ 9), group 9 (9 < yr. ≤ 10), group 10 (10 < yr. ≤ 11), and group 11 (11 < yr. ≤ 12).

### Anesthesia protocol

No children were pre-medicated. Children routinely fasted from food for ≥6 h and from drink for ≥4 h before surgery. Peripheral intravenous access was established for all patients in the hospital ward. In the operating room, a continuous monitor (S/5TM monitor; GE Healthcare, Germany) was used for noninvasive blood pressure (NIBP), pulse oxygen saturation (SpO_2_), heart rate (HR), and electrocardiogram (ECG) monitoring. After oxygen inhalation via a facemask was initiated, general anesthesia was induced by the continuous intravenous injection of 3 mg/kg of propofol (1% propofol in a medium-chain/long-chain triglyceride emulsion; Fresenius Kabi Deutschland Gmbh, Germany) for over 30 s, followed by an observation period of 4 min. Routine oxygen inhalation via the facemask was continued during propofol administration, and the lower jaw was gently raised as needed to assist airway management and maintain a blood oxygen saturation of ≥97%. At the end of the observation period, 2 mg/kg of propofol (Fresenius Kabi Deutschland Gmbh, Germany), 2 μg/kg of fentanyl (Yichang Humanwell Pharmaceutical Co., Ltd., Hubei Province, China), and 0.5 mg/kg of rocuronium (Hameln Pharmaceuticals Gmbh, Germany) were administered for routine induction. The patient was intubated or received a laryngeal mask after a sufficient depth of anesthesia was achieved.

### Data acquisition

BIS (Aspect Medical System, USA) was simultaneously monitored for all patients. The skin over the forehead was cleaned, and the BIS electrode was then placed according to the manufacturers instructions by the anesthesiologist. The BIS monitor requires a self-test before it begins to function. Propofol was injected after signal stabilization, which indicated the BIS value in the absence of anesthesia. The baseline values were recorded immediately after propofol injection was initiated (0 s). Subsequently, values were recorded at 30, 40, 50, 60, 90, 120, 180, and 240 s after the injection.

For all patients, the same anesthesiologist placed all electrodes and administered propofol. Moreover, data for all patients were recorded by the same individual.

### Statistical analysis and data handling

The sample size of 15 cases per group in the present study was generally similar, but not identical, to that of previously published reports [7, 8].

BIS values were measured at nine time points in the 11 age groups. IBM SPSS 21.0 (SPSS Inc., Chicago, IL, USA) was used for data analysis. Normally distributed numerical data are expressed as means ± standard deviations (SDs;$\overline{\mathrm{x}}\pm \mathrm{s}$). Repeated measures ANOVA was used to analyze BIS values at multiple time points in groups. A post-hoc Bonferroni test was used to compare differences at same time points in different age groups and at different time points in same groups. A *p*-value of < 0.05 was considered statistically significant. Data analysis was independently performed by two data scientists.

## Results

From the 165 initially recruited patients, eight were excluded because the electrodes fell off during the observation period; 157 patients were thus included in the analyses. The patient data are presented in Table 1.

**Table 1:** Patient characteristics of 11 groups

Groups	1	2	3	4	5	6	7	8	9	10	11	Total
Number of patients	15	15	15	15	13	15	15	15	13	12	14	157
Male/Female	13/2	14/1	13/2	10/5	7/6	10/5	12/3	13/2	6/7	9/3	9/5	116/41
Type of surgery												
Urology	11	10	6	7	9	5	5	1		3	5	62
Orthopaedics	1	3	9	6	3	7	10	9	9	9	7	73
Other	3	2		2	1	3		5	4		2	22

group 1 (1 < yr. ≤ 2), group 2 (2 < yr. ≤ 3), group 3 (3 < yr. ≤ 4), group 4 (4 < yr. ≤ 5), group 5 (5 < yr. ≤ 6), group 6 (6 < yr. ≤ 7), group 7 (7 < yr. ≤ 8), group 8 (8 < yr. ≤ 9), group 9 (9 < yr. ≤ 10), group 10 (10 < yr. ≤ 11), and group 11 (11 < yr. ≤ 12)

**Fig. 1:**
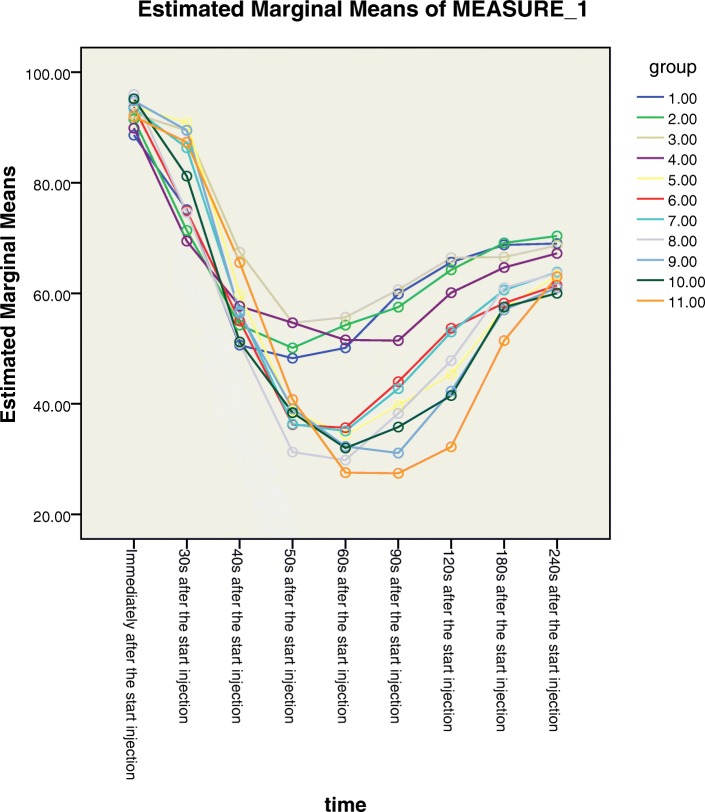
Bispectral index (BIS) values according to the patient age and time point of measurement after propofol injection in children. Age groups**:**group 1 (1 < yr. ≤ 2), group 2 (2 < yr. ≤ 3), group 3 (3 < yr. ≤ 4), group 4 (4 < yr. ≤ 5), group 5 (5 < yr. ≤ 6), group 6 (6 < yr. ≤ 7), group 7 (7 < yr. ≤ 8), group 8 (8 < yr. ≤ 9), group 9 (9 < yr. ≤ 10), group 10 (10 < yr. ≤ 11), and group 11 (11 < yr. ≤ 12)

Repeated measures ANOVA showed that BIS values significantly differed among the nine time points (*P* < 0.01) as well as the different age groups (*P* < 0.01) after propofol administration. The post-hoc Bonferroni test showed a difference in BIS values between groups 1–4 (1 < yr. ≤ 5) and groups 5–11(5 < yr. ≤ 12) (Fig. 1).

**Fig. 2:**
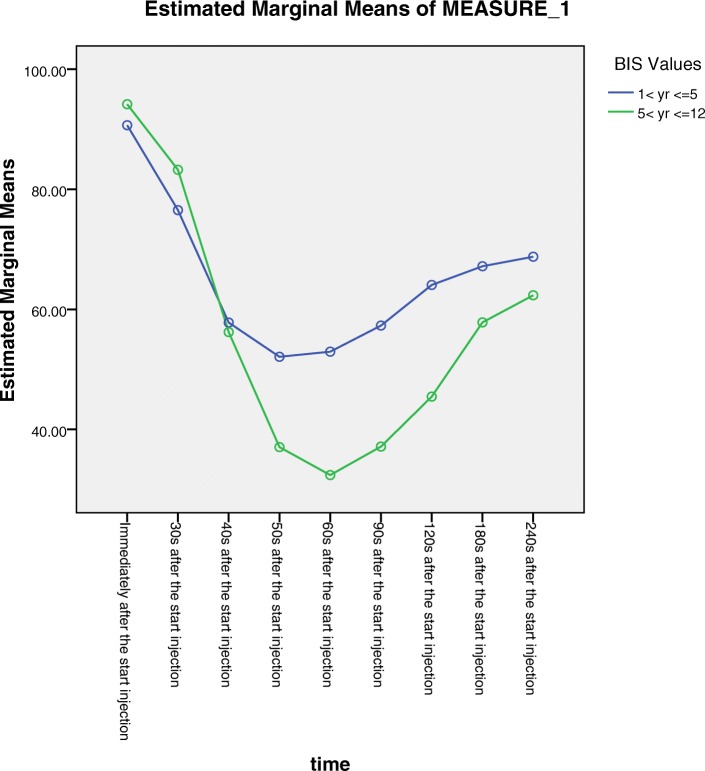
Changes in bispectral index (BIS) values over time in group 1 < yr. ≤ 5 and group 5 < yr. ≤ 12. Group 1 < yr. ≤ 5: the 60 paediatric patients in groups 1–4 (group 1: 1 < yr. ≤ 2; group 2: 2 < yr. ≤ 3; group 3: 3 < yr. ≤ 4; group 4: 4 < yr. ≤ 5), who were combined because of the lack of significant differences in BIS values. Group 5<yr. ≤ 12: the 97 paediatric patients in groups 5–11 (group 5: 5 < yr. ≤ 6; group 6: 6 < yr. ≤ 7; group 7: 7 < yr. ≤ 8; group 8: 8 < yr. ≤ 9; group 9: 9 < yr. ≤ 10; group 10: 10 < yr. ≤ 11; group 11: 11 < yr. ≤ 12), who were combined because of the lack of significant differences in BIS values

The patients were reclassified for further analysis: group 1 < yr. ≤ 5, which included the 60 patients in groups 1–4, and group 5 < yr. ≤ 12, which included the 97 patients in groups 5–11. Table 2 presents the BIS values at each time point for group 1 < yr. ≤ 5 and group 5 < yr. ≤ 12. In both groups, the baseline BIS values (immediately after the start of injection; 0 s) differed significantly from the values at the subsequent eight time points, from 30 to 240 s (*p* < 0.01). The BIS values between the two groups were significantly different at each of the following time points: 50, 60, 90, 120, 180, and 240 s (*p* < 0.05). The BIS values in group 5 < yr. ≤ 12 were significantly lower than those in group 1 < yr. ≤ 5 at each time point from 50 to 250 s (*p* < 0.05). The minimum observed BIS values for group 1 < yr. ≤ 5 and group 5 < yr. ≤ 12 were recorded at 60 s: 49 ± 17 and 35 ± 14, respectively. In group 5 < yr. ≤ 12, BIS values at 40, 50, and 60 s were significantly different from one another (*p* < 0.01); group 1 < yr. ≤ 5 did not feature significant differences among these time points (*p* > 0.05). Figure 2 shows the changes in BIS values over time in groups 1 < yr. ≤ 5 and 5 < yr. ≤ 12. BIS values in the two patient groups exhibited a rapid decrease to their minimum values from 0 to 60 s, followed by an obvious trend towards an increase. Changes in BIS values were more obvious in group 5 < yr. ≤ 12 than in group 1 < yr. ≤ 5, with the former demonstrating a precipitous decrease between 40 and 60 s.

**Table 2:** BIS values at each time point in Group 1 < yr. ≤ 5 and Group 5 < yr. ≤ 12 $\left(\overline{\mathrm{x}}\pm \mathrm{s}\right)$

time/s	BIS	
	Group 1 < yr. ≤ 5	Group 5 < yr. ≤ 12
0	91 ± 7	94 ± 5
30	75 ± 20^*^	82 ± 21^*^
40	57 ± 21^*cd^	58 ± 25^*e^
50	52 ± 18^*ac^	37 ± 16^*af^
60	49 ± 17^*ac^	35 ± 14^*af^
90	55 ± 13^*acd^	37 ± 11^*af^
120	63 ± 8^*ad^	46 ± 14^*a^
180	66 ± 5^*ad^	57 ± 8^*ae^
240	67 ± 5^*ad^	62 ± 5^*ae^

^a^ *P* < 0.05, values of BIS were compared between the group 1 < yr. ≤ 5 and the group 1 < yr. ≤ 2 at the same time point

*P < 0.01, BIS values with superscript * compared with BIS values at 0 s time point

^c^ *P* < 0.05, BIS values with superscript c compared with BIS values at the other time point of group 1 < yr. ≤ 5

^d^ *P* < 0.05, BIS values with superscript d compared with BIS values at the other time point of group 1 < yr. ≤ 5

^e^ *P* < 0.05, BIS values with superscript e compared with BIS values at the other time point of group 5 < yr. ≤ 12

^f^ *P* < 0.05, BIS values with superscript d compared with BIS values at the other time point of group 5 < yr. ≤ 12

### Time points

0: immediately after the start of injection.

30, 40, 50, 60, 90, 120, 180, and 240 s after the start of propofol injection.

## Discussion

In this study, BIS value changes were studied in children aged 1–12 years, who were grouped according to age, under propofol anesthesia. We found that BIS values showed a similar trend at each time point after propofol injection in the 11 groups, but BIS values differed according to age groups. Our findings differed from those reported previously.

The pediatric nervous system differs from that of adults in that it is characterized by rapid development; hence, typical pediatric EEGs are more variable than adult EEGs. It has been reported that EEG signals of children mature and stabilize at approximately 12 years of age [9]. Furthermore, while previous reports have studied the relationship between BIS values and propofol after age grouping, most adopted the traditional age grouping method, with the youngest patients being over 3 years old [4, 8, 10, 11]. While relevant studies on children younger than 3 years of age have been conducted, the age classification that they employed was inconsistent and lacked an appropriate justification [1, 7]. Changes in EEG signals during the growth and development of young children occur in a short period of time; BIS values may thus feature a similarly significant change and thereby impact the accuracy of BIS monitoring. This study therefore divided children aged 1–12 years into 11 groups with one-year intervals to determine whether BIS values differed among the groups stratified by age.

As the utility of BIS for monitoring the sedative depth of propofol anesthesia in children has been established, propofol is widely used for both sedation and general anesthesia in children [12]. However, BIS monitoring is easily disrupted by the administration of other drugs, especially muscle relaxants. Because BIS features frontal electromyography (EMG) as a weighted parameter, the frontal EMG overlaps with the EEG at a frequency of 30–50 Hz, and the interference of frontal EMG can thus be excluded at frequencies lower than 47 Hz. However, after the application of muscle relaxants, BIS may decrease due to the drop in the components of the frontal EMG, and the monitoring of sedation depth may be disrupted. The BIS value can reportedly reduce to 33 when muscle relaxants are administered to patients who are fully awake [13]. Whether opioids have an impact on the BIS value remains controversial: although many studies have reported that opioids do not affect BIS values [14, 15], Minto and colleagues [16] have described age-dependent EEG modification when remifentanil was administered to adults and found that changes in BIS values with an attendant loss of verbal command or eyelash reflex were more common in patients who received remifentanil combined with propofol than propofol alone [17, 18]. Therefore, this study administered a single propofol to eliminate the effect of the combined drugs on BIS value and improve the accuracy of the research results.

Previous literature has shown that BIS values for children under propofol anesthesia are significantly associated with age [1]; however, prior studies have only shown that BIS accuracy is better in children over the age of 1 year. In this study, the results of detailed age grouping indicated that BIS values in the 11 groups showed a similar trend in BIS: an initial decrease followed by an increase. However, the changes of BIS values at the same time points differed significantly across age. Moreover, we identified that such significant differences could be identified between two composite groups: 1–4 and 5–11. The results suggest that age persists as an important factor, affecting BIS in children over 1 year of age, and anesthesiologists must pay attention to this difference when monitoring sedation depth.

The minimum BIS values of 49 ± 17 and 35 ± 14 in group 1 < yr. ≤ 5 and group 5 < yr. ≤ 12, respectively, were observed at 60 s. Generally consistent with reports of the peak effect time being at 1.6 min (1–2.4 min) after the single-bolus intravenous injection of propofol [19]. From this finding we presume that BIS is sensitive to changes in blood propofol concentration and similar result had been reported in the previous literature [20]. The change of BIS values in group 5 < yr. ≤ 12 was more rapid than that in group 1 < yr. ≤ 5; the former featured a precipitous decrease between 40 and 60 s, and the change in BIS over the same period was not statistically significant in group 1 < yr. ≤ 5. In addition, the BIS values at each time point from 50 to 240 s showed consistent statistically significant differences between group 1 < yr. ≤ 5 and group 5 < yr. ≤ 12; the BIS values in group 5 < yr. ≤ 12 were all lower than those in group 1 < yr. ≤ 5. This result is consistent with those of previous reports that found BIS values in older age groups to be lower than those in younger age groups [7, 8]. These findings imply that anesthesiologists should appropriately reduce the single injection dose of propofol for children over 5 years old in order to avoid BIS values of less than 40.

There were several limitations of this study that were important to note. First, this study did not provide hemodynamics data at each time point. The current study was designed to observe the change of BIS value after single dose of propofol injection and the 10-s interval between each observation time point was narrow, it may be difficult to measure non-invasive blood pressure at each time points and we also considered that even if we did measure it, Moreover, cuff blood pressure measurement may be considered as one of stimulations during the observation period which may interfere with the sedative state of the child. But heart rate and pulse oxygen saturation were continuously monitored during the whole procedure. Secondly, the current study did not provide information of clinical parameters at each time points, the correlation between BIS value and clinical consciousness was therefore not evaluated in this study. This was the initial observational study for BIS value and propofol injection in different age groups and the correlation BIS values and clinical signs of sedative should be assessed in our future studies.

## Conclusions

Our study indicates that BIS values showed a close relationship to two age groups: 1 < yr. ≤ 5 and 5 < yr. ≤ 12 during the administration of propofol anesthesia. BIS values of the older age group were lower than those of the younger age group at the same time points. This study suggests that attention should be paid to changes in children younger and older than 5 years when BIS monitoring is applied to children aged 1–12 years.

## Data Availability

We declared that materials described in the manuscript, including all relevant raw data, will be freely available to any scientist wishing to use them for non-commercial purposes, without breaching participant confidentiality by contacting the corresponding author.

## Abbreviations

BIS: Bispectral index
EEG: Electroencephalogram
